# Splicing: is there an alternative contribution to Parkinson’s disease?

**DOI:** 10.1007/s10048-015-0449-x

**Published:** 2015-05-16

**Authors:** Valentina La Cognata, Velia D’Agata, Francesca Cavalcanti, Sebastiano Cavallaro

**Affiliations:** Institute of Neurological Sciences, Italian National Research Council, Via Paolo Gaifami 18, 95125 Catania, Sicily Italy; Institute of Neurological Sciences, Italian National Research Council, 87050 Piano Lago di Mangone, Cosenza, Calabria Italy; Department of Biomedical and Biotechnological Sciences, Section of Human Anatomy and Histology, University of Catania, Catania, Italy

**Keywords:** Parkinson’s disease, Alternative splicing, PD genes, mRNA splice transcripts, Protein isoforms

## Abstract

Alternative splicing is a crucial mechanism of gene expression regulation that enormously increases the coding potential of our genome and represents an intermediate step between messenger RNA (mRNA) transcription and protein posttranslational modifications. Alternative splicing occupies a central position in the development and functions of the nervous system. Therefore, its deregulation frequently leads to several neurological human disorders. In the present review, we provide an updated overview on the impact of alternative splicing in Parkinson’s disease (PD), the second most common neurodegenerative disorder worldwide. We will describe the alternative splicing of major PD-linked genes by collecting the current evidences about this intricate and not carefully explored aspect. Assessing the role of this mechanism on PD pathobiology may represent a central step toward an improved understanding of this complex disease.

## Introduction

The flow of genetic information from DNA to RNA to protein has traditionally been considered the central dogma of molecular biology. Additional steps of regulation are currently well known, greatly expanding this simplistic framework and revealing the complex network that controls gene expression [1]. One of these steps is represented by alternative splicing (AS), whereby a single gene gives rise to multiple messenger RNA (mRNA) transcripts and protein isoforms with different functional properties [1]. It is estimated that 94 % of human protein-coding genes are alternatively spliced [2, 3], and the main site of alternative splicing events is the central nervous system [4, 5].

**Fig. 1:**
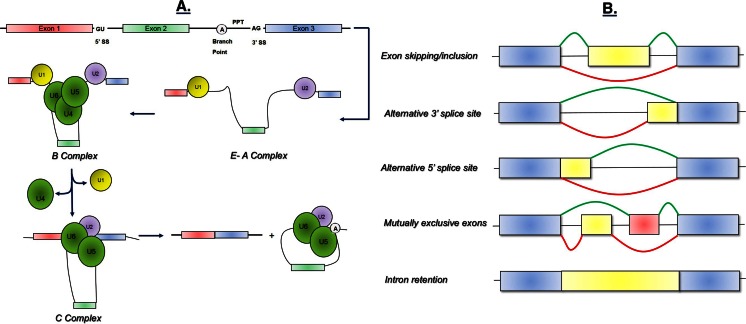
The alternative splicing mechanism. **a** Four main conserved DNA sequence motifs allow the splicing mechanism: the donor splice site GU (*5*′ *SS*), the acceptor splice site AG (*3*′ *SS*), the lariat branch point (*A*) located upstream of the acceptor site and the polypyrimidine tract (*PPT*) placed between the acceptor site and the branch point. The splicing machinery includes mainly five spliceosomal uridine-rich small nuclear ribonucleoproteins (snRNPs) (*U1*, *U2*, *U4*, *U5*, and *U6*) and further auxiliary RNA binding proteins. During the first step of spliceosome assembly, U1 snRNP base pairs with the 5′ splice site of the pre-mRNA (*E complex*), whereas U2 base pairs with the branch point (*A complex*). Then the tri-snRNP complex U4, U5, and U6 associates with the forming spliceosome (*B complex*), and both U1 and U4 are ejected. This allows U6 to replace U1 at the 5′ splice site (*C complex*) and leads to a U6–U2 interaction that gets close together the 5′ splice site and the branch point, allowing for a transesterification step. At the end, U5 brings near the two exons, joining them through a second transesterification reaction. **b** Five major alternative splicing events are currently known: exon skipping/inclusion, use of alternative 3′ splice site, use of alternative 5′ splice site, mutually exclusive exons, and intron retention. In *blue*, are represented the constitutive exons. *Yellow* and *red* represent the alternatively spliced exons. The splicing events rely on the interplay between the constitutive splicing motifs, the splicing regulatory sequences, the RNA secondary structures, the components of the spliceosome, and further auxiliary RNA-binding proteins. However, how the spliceosome decides which exons to include remains currently not clear

The alternative splicing process consists in the removal of the intronic regions from the RNA primary transcript and simultaneous assembly of the exonic regions in different combinations to form a mature mRNA, which is then polyadenilated, exported to the cytoplasm, and translated into protein. The accuracy and efficiency of pre-mRNA splicing process depend on a range of constitutive DNA sequence motifs: the donor and the acceptor splice sites, the lariat branch point, the polypyrimidine tract, and splicing enhancers and silencers (Fig. 1, panel a). These motifs are recognized by a large macromolecular splicing machinery (called the spliceosome), which models the pre-mRNA while RNA polymerase II synthesizes it in the nucleus. The splicing machinery includes five spliceosomal uridine-rich small nuclear ribonucleoproteins (snRNPs) (U1, U2, U4, U5, and U6) and several non-snRNP protein splicing factors such as the serine/arginine (SR)-rich protein family and hnRNP proteins [6, 7]. The splicing reaction relies on two transesterification steps that occur within the highly dynamic splicing machine. The stepwise molecular mechanisms of the splicing reaction are detailed in Fig. 1 (panel a).

Alternative splicing works as an on–off switch in gene expression. It affects the expression levels, stability, half-life [via the nonsense-mediated mRNA decay (NMD)], and localization of the RNA messengers. It has also the potential to generate several protein isoforms with different biological properties, protein–protein interactions, subcellular localization, signaling pathway, or catalytic ability. During the last years, great efforts have been made to decipher the intricate alternative splicing code. Five major alternative splicing events (i.e., cassette exons, use of alternative acceptor and/or donor sites, intron retention, and mutually exclusive exons) have been described up to now and are detailed in Fig. 1 (panel b) [2, 8]. However, how the spliceosome recognizes alternative exons and decides which exons to include remains not fully understood. Undoubtedly, there is more diversity in splice transcript variants than in protein isoforms. Although this is still not clear, different variants encode the same protein, but probably translate it with different efficiencies [9].

The finely tuned splicing regulatory network can easily undergo alterations. An aberrant alternative splicing may arise from changes in regulatory sequences required for correct pre-mRNA processing (the so-called *cis*-acting mutations), as well as from mutations that affect components necessary for splicing regulation (*trans*-acting mutations). *Cis*- and *trans*-splicing aberrations represent direct causative agents of disease or more subtle contributions to the determinants of disease susceptibility or modulators of disease severity. An extensive range of neurological diseases has been already associated to both splicing defects, including Alzheimer’s disease, retinitis pigmentosa, spinal muscular atrophy, muscular dystrophy, neurofibromatosis, and fragile X-associated tremor/ataxia syndrome [10–12, 1, 13]. In this broad neurological disorder scenario, the relevance of alternative splicing in Parkinson’s disease (PD) is not still clear, and the splicing mechanisms that regulate PD-related genes remain mostly unknown.

Here, we provide an updated overview of the current knowledge about the impact of alternative splicing on Parkinson’s disease. Firstly, we will take into account the most common PD-related genes “one by one” by analyzing their alternative transcripts currently known and their involvement in this disease. Then, we will describe the few studies that have globally analyzed the changes of splice variant expression in PD patients through genome-wide RNA expression approaches. Finally, we will briefly describe the current evidences about the alternative splicing modulation in PD through noncoding RNAs [microRNA (miRNA) and long noncoding RNA (lcnRNA)].

## Genetics of Parkinson’s disease

PD is the second most common neurodegenerative disorder worldwide, characterized by resting tremor, bradykinesia, stiffness of movement, and postural instability. These symptoms are derived from the progressive loss of neurons from the *substantia nigra pars compacta*, coupled with an accumulation of intraneuronal aggregates called Lewy bodies.

Despite significant progresses in the understanding of PD pathogenesis, the exact etiology of PD remains unknown. Over the past 15 years, an even more detailed knowledge of the genetic factors that contribute to PD has emerged through different research strategies [14, 15]. Linkage mapping analysis, genome-wide association studies (GWAS), and next-generation sequencing technologies are revealing an increasing number of locus and genes strongly linked to either autosomal dominant (*SNCA*-*PARK1*, *LRRK2*-*PARK8*, *VPS35*-*PARK17*, and *GBA*), or typical recessive (*PARKIN*-*PARK2*, *PINK1*-*PARK6*, and *DJ1*-*PARK7*) and atypical recessive (*ATP13A2*-*PARK9*, *PLA2G6*-*PARK14*, and *FBXO7*-*PARK15*) or X-linked (*ATP6A2* and *TAF1*) forms of disease. For the sake of completeness, we mention here further monogenic loci, not confirmed genes, or risk factor genes (i.e., *PARK3*, *UCHL1*, *PARK10*, *GIGYF2*, *PARK12*, *HTRA2*, *PARK16*, *EIF4G1*, *DNAJ*, *HLA*-*DR*, *GAK*-*DGKQ*, *SYNJ1*, and *GBAP1*) [15–17]. Furthermore, a large-scale meta-analysis of genome-wide association data is revealing a wide range of additional loci having genome-wide significant association [18]. However, we will overlook their discussion because of the few data in the literature regarding their splicing regulation in pathological conditions.

In the next paragraphs, we will describe the alternative spliced mRNA variants of PD genes and the current scientific data demonstrating their involvement in PD pathogenesis. For a more complete picture, we have also added some further implicated genes (*SRRM2*, *MAO*-*B*, *SNCAIP*, *MAPT*, and *GBA*), indicated as other PD-related genes, which are not directly causative genes, but whose splicing regulation seems to be altered in PD states.

### Autosomal dominant PD genes

#### SNCA

Alpha-synuclein, encoded by *SNCA* gene, is a small, natively unfolded presynaptic protein linked to PD [19]. Aggregates of alpha-synuclein protein represent the neuropathological hallmark lesions of PD and constitute the major components of Lewy bodies. Genetically, mutations in *SNCA* gene were the first to be associated with PD family inheritance. Missense mutations in coding regions (Ala53Thr, Ala30Pro, and Glu46Lys), single nucleotide substitution in 3′ untranslated region (3′ UTR), and dose-dependent genomic multiplications (duplications or triplications) of the gene cause both monogenic and sporadic forms of PD [20, 19, 21]. Some point mutations in splice donor sites have also been reported (IVS2 + 9A > C) [22].

**Fig. 2:**
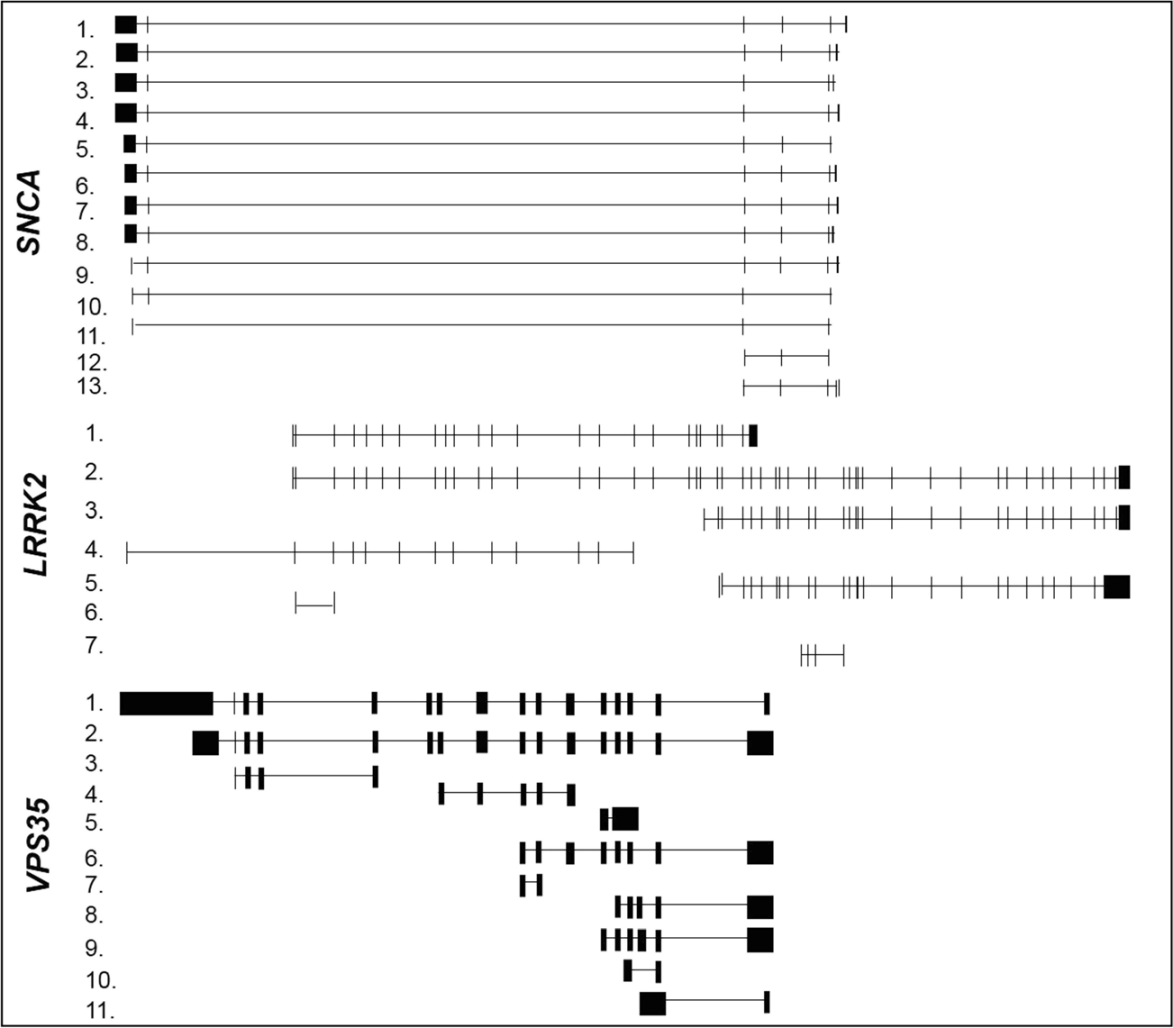
Structures of the alternative splicing variants of human dominant PD genes. Structures of the described mRNA splicing variants are represented in the figure as reported in Ensembl library (http://www.ensembl.org/index.html). On the *left*, each variant is indicated with a number corresponding to that indicated in Table 1. *LRRK2* gene is illustrated in 5′-3′ sense, while *SNCA* and *VPS35* genes are illustrated in antisense corresponding to their 3′-5′ sense transcription

*SNCA* gene maps to chromosome 4q22.1 and contains six exons spanning about 114 kb [21]. The set of mRNAs produced by *SNCA* gene includes the *full*-*length* transcript, commonly known as SNCA-140 from the amino acidic length of the encoded protein, and corresponds to SNCA-001, SNCA-002, SNCA-003, SNCA-006, and SNCA-008 mRNAs from Ensembl library (Table 1 and Fig. 2). Further additional splicing variants, known as SNCA-126, SNCA-112, and SNCA-98 and corresponding to (i) SNCA-004, SNCA-203, SNCA-201, (ii) SNCA-005, SNCA-202, and (iii) SNCA-010, respectively, are generated by in-frame excision of exons 3, 5, or both (Table 1 and Fig. 2). Two additional splice variants (SNCA-009 and SNCA-007) are generated from an inner transcription start and encode proteins of 115 and 97 amino acids, respectively (Table 1 and Fig. 2). SNCA-140, SNCA-126, and SNCA-112 are expressed in a broad spectrum of human tissues, while SNCA-98 seems to be a brain-specific splice variant with varying expression levels in different areas of fetal and adult brain [23].

**Table 1:** Alternative splice variants of human autosomal dominant PD genes

Gene name	Transcript number	Ensembl name	Genbank accession number	Protein length
*SNCA*	1.	SNCA-003	NM_001146055	140 aa
	2.	SNCA-202	NM_007308	112 aa
	3.	SNCA-203	–	126 aa
	4.	SNCA-201	–	126 aa
	5.	SNCA-005	–	112 aa
	6.	SNCA-001	NM_001146054	140 aa
	7.	SNCA-002	NM_000345	140 aa
	8.	SNCA-008	–	140 aa
	9.	SNCA-006	–	140 aa
	10.	SNCA-004	–	126 aa
	11.	SNCA-010	–	98 aa
	12.	SNCA-009	–	115 aa
	13.	SNCA-007	–	67 aa
*LRRK2*	1.	LRRK2-002	–	1271 aa
	2.	LRRK2-004	NM_198578	2527 aa
	3.	LRRK2-005	–	207 aa
	4.	LRRK2-001	–	521 aa
	5.	LRRK2-003	–	No protein
	6.	LRRK2-006	–	No protein
	7.	LRRK2-007	–	No protein
*VPS35*	1.	VPS35-001	NM_018206	796 aa
	2.	VPS35-002	–	48 aa
	3.	VPS35-011	–	No protein
	4.	VPS35-012	–	No protein
	5.	VPS35-006	–	No protein
	6.	VPS35-003	–	No protein
	7.	VPS35-010	–	No protein
	8.	VPS35-005	–	47 aa
	9.	VPS35-008	–	41 aa
	10.	VPS35-007	–	No protein
	11.	VPS35-004	–	No protein

Gene name, Ensembl transcript names, GenBank accession numbers, and relative encoded amino acidic protein length of splice variants are reported in the table. Number in the column “Transcript number” identifies the transcript in Fig. 2

The expression profile of SNCA-140, SNCA-126, SNCA-112, and SNCA-98 splice variants is different in the various brain areas under normal and pathological states. Compared to healthy controls, in PD *frontal cortex*, all these four transcripts are overexpressed, with significant upregulation of SNCA-126 [24]. In PD *substantia nigra*, only the three shorter transcripts have been observed significantly overexpressed [25, 26], while higher SNCA-112 and SNCA-98 levels are also present in the *cerebellum* [25]. Different expression profiles of *SNCA* variants also occur in other forms of neurodegenerative disorders. Both SNCA-140 and SNCA-126 downregulation and SNCA-98 overexpression have been reported in dementia with Lewy bodies and Alzheimer’s disease, while SNCA-112 is upregulated in dementia with Lewy bodies and downregulated in Alzheimer’s disease [27, 28, 24].

Some interesting data emerge on SNCA-112 variant. An association between PD risk-associated single nucleotide polymorphisms (SNPs) within the 3′ region of *SNCA* gene and higher SNCA-112 ratio level has been observed in about 100 of frontal cortex samples. These data reveal the *cis*-regulatory effect of these mutations on splicing mechanism [29]. The expression of SNCA-112 is also abundantly induced by some parkinsonism mimetics (MPP+, rotenone) and related oxidants [30]. However, the reason for these effects remains unclear.

In addition to splice variants, specific RNA transcript isoforms of *SNCA* with an extended 3′ untranslated region have been described and appear selectively linked to pathological processes [31]. However, this review is focusing only on the mRNA splice variants; thus, their discussion will be omitted.

The 140 amino acid isoform is a small protein with a molecular weight of 14.5 kDa. It is composed of three distinct regions: (1) an amino terminus containing amphipathic helices conferring the propensity to bind membranes; (2) a central hydrophobic region, the so-called non-Ab component (NAC), which confers the b-sheet potential; and (3) an acid glutamatergic carboxyl terminus that is highly negatively charged and prone to be unstructured [19]. Structural changes in the shorter splicing isoforms can be predicted as a result of exon skipping events. SNCA-126-predicted isoform shows interruption of the N-terminal protein–membrane interaction domain [32]; SNCA-112 is significantly shorter in the unstructured C-terminal [32], while SNCA-98 isoform results in a truncated protein consisting almost only of the central region containing NAC [23]. Recently, a lower aggregation propensity of the shorter isoforms has been demonstrated in vitro [33]. In addition, morphology studies by using electron microscopy have shown straight fibrils for SNCA-140, shorter fibrils mostly arranged in parallel arrays for SNCA-126, and circular structures for SNCA-98 [33]. These data open new insights regarding the formation of Lewy bodies induced by alpha-synuclein.

Numerous functions of alpha-synuclein have been proposed, counting molecular chaperone, regulator of dopamine uptake and homeostasis, inhibitor of phospholipase D2, downregulator of p53 pathway [32], and promoter of the SNARE-complex assembling [34]. Unfortunately, nothing is known about the specific pathophysiological roles of each alpha-synuclein isoform and their relative posttranslational modifications (i.e., phosphorylations, nitration, sumoylation, oxidation, glycosylation, cleavage, and ubiquitination), which are known to play a key role in SNCA functions and regulation [32].

#### LRRK2

*LRRK2* encodes for leucine-rich repeat kinase 2 (or dardarin), which is a large 2527 amino acid multidomain protein. The protein consists of multiple conserved well-defined domains including a small GTPase-like domain (Ras of complex proteins or ROC), a domain of unknown function termed the C-terminal of ROC (COR), a kinase domain, as well as several protein interaction domains [e.g., the leucine-rich repeat (LRR), the WD40 domain, the ankyrin repeat domain, and the armadillo repeat region]. The precise physiological function of LRRK2 is unknown. However, LRRK2 seems implicated in different cellular functions as neurite outgrowth, cytoskeletal maintenance, vesicle trafficking, and autophagic protein degradation [35].

The *LRRK2* gene spans a genomic region of 144 kb, with 51 exons, and harbors the most common mutations linked to both autosomal dominant inherited late-onset and sporadic PD. The missense mutations known so far are spread over the whole *LRRK2* gene and affect all functional domains. Some mutations have much higher frequencies than others, such as Gly2019Ser and mutations altering codon Arg1441, respectively, in the kinase and ROC domains. In addition, several unclear pathogenic mutations affecting splice sites have been observed (IVS19 + 5_8delGTAA, IVS25-8delT, IVS27-9C > T, IVS30-6C > T, IVS31 + 3A > G, IVS32 + 14G > A, IVS33 + 6 T > A, IVS37-9A > G, IVS38 + 7C > T, IVS46-14 T > A, and IVS46-8delT) [36, 22, 37–43].

In addition to the full-length transcript (LRRK2-004), further *LRRK2* shorter transcripts are deposited in Ensembl library (Table 1 and Fig. 2). Despite the existence of these transcripts, there are currently no data analyzing the splicing profile of this gene in PD states. Recently, a gene expression and splicing analysis of the *LRRK2* locus have been carried on [44]. Both exon array and RT-PCR methods confirm the existence of an isoform with spliced out exons 32–33 in the *substantia nigra* and an isoform with exon 32 alone spliced out in the *occipital cortex*, *medulla*, and *cerebellum* of healthy humans [44].

Further evidences on *LRRK2* splicing have been observed by Giesert and collaborators [45], who have conducted a study in various brain regions and organs from adult mice. In this regard, it should be considered that *LRRK2* is highly conserved in human and mouse and that several transgenic animal models have been created. Giesert et al. [45] have identified two *LRRK2* splice variants: one with skipped exon 5, primarily expressed in astrocytes, and another truncated variant terminating with an alternative exon 42a barely detectable in the microglia but highly expressed in neurons and astrocytes. Protein-structure predictions reveal that the loss of exon 5 may generate a smaller protein with changed affinity of binding partners, while the alternative exon 42a may lead to changes of its enzymatic activity. In addition, the protein-interaction domain WD40 would also be absent in such truncation. Interestingly, the deletion of this domain in the Zebrafish LRRK2 ortholog (zLRRK2) causes parkinsonism-like phenotype including loss of dopaminergic neurons in diencephalon and locomotion defects [46]. Further studies will need to assess the involvement of *LRRK2* alternative splice variants in PD.

#### VPS35

In 2011, two groups reported the identification of the same missense mutation (p.Asp620Asn) in the vacuolar protein sorting 35 (*VPS35*) gene as a novel cause of autosomal dominant PD [47, 48]. *VPS35* was the first PD gene found by a direct whole exome sequencing in large families of Austrian and Swiss origins. An in-depth sequence analyses of all coding, noncoding, and exon–intron boundaries *VPS35* genetic regions have been performed in a large well-characterized cohort of Lewy body disorders, including PD patients, PD with dementia, and dementia with Lewy bodies [49]. In addition to three novel missense mutations, silent and intronic variations, predicted to activate cryptic splice sites, have been observed in the patient’s group but not in controls. However, the pathogenicity of these mutations was not completely conclusive since these mutations were not supported by segregation analysis in family relatives [49].

Various spliced transcript variants of this gene are reported in Ensembl library (Table 1 and Fig. 2), but the majority of them are processed for degradation and do not encode proteins.

### Autosomal recessive PD genes

#### Early-onset typical PD genes

##### *PARK2*

Mutations in *PARK2* gene (also known as *PARK2 parkin RBR E3 ubiquitin*-*protein ligase*) are the most common cause (50 % of cases) of autosomal recessive juvenile parkinsonism (AR-JP), a form of early-onset parkinsonism characterized by good and prolonged response to levodopa and a benign, slow course. *PARK2* mutations also explain ~15 % of the sporadic cases with onset before 45 [50, 51] and act as susceptibility alleles for late-onset forms of Parkinson’s disease (2 % of cases) [52]. Along with about 200 mutations currently identified in *PARK2* coding region, several point mutations in splice acceptor or donor sites (introns 1, 6, 7, 10, 12, 13, and 16) have been identified in PD patients [53–57, 22, 58, 59].

**Fig. 3:**
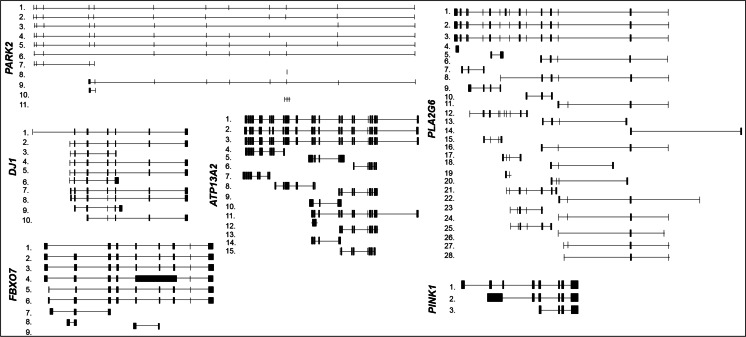
Structures of the alternative splicing variants of human recessive PD genes. Structures of the described mRNA splicing variants are represented in the figure as reported in Ensembl library (http://www.ensembl.org/index.html). On the *left*, each variant is indicated with a *number* corresponding to that indicated in Table 2. All transcripts are illustrated in 5′-3′ sense, except *PARK2*, *ATP13A2*, and *PLA2G6* genes, which are illustrated in antisense corresponding to their 3′–5′ sense transcription

*PARK2* gene spans more than 1.38 Mb of genomic DNA in the long arm of chromosome 6 (6q25.2–q27) and contains 12 exons, which are alternatively spliced to produce at least 11 different splicing variants (Table 2 and Fig. 3) [59]. The full-length *PARK2* transcript (PARK2-004) encodes a protein of 465 amino acids (parkin) [60, 61, 59] acting in numerous molecular pathways (protein turnover, stress response, mitochondrial homeostasis, mitophagy, mitochondrial DNA stability, metabolism, cell growth, and survival) [62]. Multiple parkin isoforms likely arising from *PARK2* splicing variants have been observed in different brain areas through Western blot studies [9].

**Table 2:** Alternative splice variants of human autosomal recessive PD genes

Gene name	Transcript number	Ensembl name	Genbank accession number	Protein length
*PARK2*	1.	PARK2-004	NM_004562	465 aa
	2.	PARK2-005	NM_013987	437 aa
	3.	PARK2-006	NM_013988	316 aa
	4.	PARK2-001	–	274 aa
	5.	PARK2-003	–	274 aa
	6.	PARK2-007	–	218 aa
	7.	PARK2-201	–	176 aa
	8.	PARK2-204	–	87 aa
	9.	PARK2-002	–	368 aa
	10.	PARK2-202	–	74 aa
	11.	PARK2-203	–	201 aa
*PINK1*	1.	PINK1-001	NM_032409	581 aa
	2.	PINK1-002	–	No protein
	3.	PINK1-003	–	No protein
*DJ1*	1.	PARK7-004	–	189 aa
	2.	PARK7-002	NM_001123377; NM_007262	189 aa
	3.	PARK7-007	–	No protein
	4.	PARK7-001	–	189 aa
	5.	PARK7-003	–	169 aa
	6.	PARK7-008	–	No protein
	7.	PARK7-005	–	189 aa
	8.	PARK7-006	–	189 aa
	9.	PARK7-009	–	No protein
	10.	PARK7-010	–	160 aa
*ATP13A2*	1.	ATP13A2-001	NM_022089	1180 aa
	2.	ATP13A2-002	NM_001141974	1158 aa
	3.	ATP13A2-005	NM_001141973	1175 aa
	4.	ATP13A2-004	–	No protein
	5.	ATP13A2-003	–	No protein
	6.	ATP13A2-010	–	191 aa
	7.	ATP13A2-007	–	398 aa
	8.	ATP13A2-014	–	258 aa
	9.	ATP13A2-009	–	321 aa
	10.	ATP13A2-006	–	No protein
	11.	ATP13A2-201	–	228 aa
	12.	ATP13A2-013	–	No protein
	13.	ATP13A2-011	–	190 aa
	14.	ATP13A2-012	–	191 aa
	15.	ATP13A2-008	–	188 aa
*PLA2G6*	1.	PLA2G6-001	NM_003560	806 aa
	2.	PLA2G6-201	NM_001004426	752 aa
	3.	PLA2G6-002	NM_001199562	752 aa
	4.	PLA2G6-025	–	No protein
	5.	PLA2G6-021	–	No protein
	6.	PLA2G6-014	–	166 aa
	7.	PLA2G6-024	–	No protein
	8.	PLA2G6-013	–	No protein
	9.	PLA2G6-026	–	168 aa
	10.	PLA2G6-015	–	120 aa
	11.	PLA2G6-010	–	99 aa
	12.	PLA2G6-023	–	226 aa
	13.	PLA2G6-009	–	No protein
	14.	PLA2G6-027	–	51 aa
	15.	PLA2G6-022	–	No protein
	16.	PLA2G6-012	–	151 aa
	17.	PLA2G6-019	–	124 aa
	18.	PLA2G6-011	–	No protein
	19.	PLA2G6-020	–	No protein
	20.	PLA2G6-008	–	No protein
	21.	PLA2G6-016	–	229 aa
	22.	PLA2G6-005	–	99 aa
	23.	PLA2G6-018	–	157 aa
	24.	PLA2G6-003	–	99 aa
	25.	PLA2G6-017	–	197 aa
	26.	PLA2G6-007	–	80 aa
	27.	PLA2G6-004	–	No protein
	28.	PLA2G6-006	–	No protein
*FBXO7*	1.	FBXO7-003	–	41 aa
	2.	FBXO7-001	NM_012179	522 aa
	3.	FBXO7-004	–	49 aa
	4.	FBXO7-005	–	No protein
	5.	FBXO7-006	–	129 aa
	6.	FBXO7-002	NM_001033024; NM_001257990	408 aa
	7.	FBXO7-007	–	54 aa
	8.	FBXO7-008	–	No protein
	9.	FBXO7-010	–	No protein

Gene name, Ensembl transcript names, GenBank accession numbers, and relative encoded amino acidic protein length of splice variants are reported in the table. Number in the column “Transcript number” identifies the transcript in Fig. 3

The extensive alternative splicing of *PARK2* is differently regulated both at transcript and protein level in tissues and cells [63, 64, 24, 65–68]. Distinct expression patterns of *PARK2* splice variants emerge in human brain regions [69] and leukocytes [68], in rat brain, in neuronal and glial cells [67], and in a wide variety of mouse tissues (brain, heart, lung, liver, skeletal muscle, kidney, and testis) [64]. At the protein level, *PARK2* protein isoforms show a differential distribution in human leukocytes [70] and aged brain [71], as well as in different rat and mouse nervous system areas (cerebral cortex/diencephalons, hippocampus, cerebellum, brainstem, striatum, spinal cord, and *substantia nigra*), peripheral tissues (heart, liver, spleen, pancreas, and kidney), and developmental stages [72–76].

Emerging evidences support the importance of *PARK2* splice variant expression changes in disease development. Differential expression of *PARK2* transcripts have been identified in the frontal cortex of Parkinson’s disease, pure dementia with Lewy bodies, common Lewy body disease, and Alzheimer’s disease patients, compared to controls [65, 24]. Particularly, two *PARK2* splicing variants are significantly overexpressed in PD [65]. Another study reports both an increase in the expression level of a parkin splice variant and a decrease of the wild type between PD patients and healthy controls [66]. The differential and disease-specific expression profiles of *PARK2* alternative splice variants suggest a role for splicing deregulation in the development of neurodegenerative disorders.

##### PINK1

Homozygous or compound heterozygous loss-of-function mutations in *PTEN*-*induced putative kinase 1* (*PINK1*) are the second most frequent cause of autosomal recessive early-onset parkinsonism. Mutation frequency varies geographically from 1 to 9 % depending on ethnic background [77]. The *PINK1* mutation spectrum involves nonsense and missense mutations, insertions, or deletions, and whole gene or single/multiple exon copy number variants located across the entire gene [78].

*PINK1* gene maps in the short arm of chromosome 1 (1p36.12), encompassing ~18 kb of genomic DNA. Its coding sequence is spread over eight exons. In addition to the full length (PINK1-001), two shorter variants exist but do not produce proteins (Table 2 and Fig. 3).

Some interesting findings emerge regarding the splicing regulation of exon 7. A 23-bp deletion disrupting the splice acceptor site of exon 7 has been detected in a sporadic parkinsonian patient, producing several aberrant mRNAs [79]. Moreover, whole exon 7 deletion and a novel U1-dependent 5′ splice-site mutation in exon 7 have been found in a large Spanish family with PD members [80].

The PINK1 protein is a putative serine/threonine kinase of 581 amino acids involved in mitochondrial response to cellular and oxidative stress [81]. It has been demonstrated that at least two isoforms are expressed in the human brain: a full-length protein of ~63 kDa and an N-terminally truncated isoform of 52 kDa [77, 82–84]. An additional isoform of approximately 45 kDa has been suggested, although it has not been extensively studied [85]. The 52-kDa isoform seems to originate by enzymatic cleavage of PARL [86]; however, the exact nature of the isoforms, the precise reason for the cleavage, and the functional roles of these three different isoforms require further studies.

##### DJ1

Mutations in the *DJ1* (also known as *PARK7*) gene are the less common cause of autosomal recessive parkinsonism (~1 % of early-onset PD) [87, 88]. A large homozygous deletion and a missense mutation (L166P) in *DJ*-*1* gene were first identified in both Italian and Dutch consanguineous families [89, 90]. Additional mutations have been collected in other PD families and include missense mutations in coding and promoter regions, frame shifts, copy number variations [91, 88], and splice site alterations [92, 93].

*DJ*-*1* gene maps to chromosome 1 (1p36.23) and includes seven exons. Several spliced transcript variants have been identified encoding the same protein (Table 2 and Fig. 3). Two shorter transcripts (the first lacking exon 4 and the second starting in an inner transcription point) encode for smaller proteins (Table 2 and Fig. 3).

The product of *DJ*-*1* gene is a highly conserved protein of 189 amino acids belonging to the peptidase C56 family [94]. It is a multifunctional protein, acting as a positive regulator of transcription, redox-sensitive chaperone, sensor for oxidative stress, and apparently protects neurons from ROS-induced apoptosis [95, 96]. In the human brain and peripheral blood, several *DJ*-*1* isoforms exist and differ on their isoelectric point (p*I*) [97–100]. The relative abundance of these different *DJ*-*1* isoforms appears to be altered in PD, and therefore, blood *DJ*-*1* isoforms have been proposed as potential biomarkers for Parkinson’s disease [101]. The different pI of each variant are believed to result from posttranslational modifications that alter the intrinsic charge of the protein [101]. Interestingly, it has been demonstrated that one of the major binding partners of DJ-1 in dopaminergic neuronal cells is the splicing factor proline/glutamine-rich (SFPQ protein) [96, 102]. SFPQ, originally identified as a polypyrimidine tract-binding protein, is part of the spliceosome C complex and is required for in vitro splicing of pre-mRNA [96, 102]. DJ-1 binding to SFPQ modulates its transcriptional activity and, therefore, tunes its effect on splicing regulation. DJ-1 mutations could reverberate on its downstream targets, including the splicing factor SFPQ and altering the splicing control.

#### Juvenile atypical PD genes

##### ATP13A2

*ATP13A2* mutations are associated with Kufor–Rakeb syndrome, a form of recessively levodopa-responsive inherited atypical parkinsonism [103]. It encodes a large protein belonging to the ATPase transmembrane transporters, and recently, it has been identified as a potent modifier of the toxicity induced by alpha-synuclein [104].

*ATP13A2* is composed of 29 exons and lies on chromosome 1 covering about 26 kb of genomic DNA. One of the first identified disease-causing mutations was a guanine-to-adenine transition in the donor splice site of exon 13, leading to the skipping of exon 13 and resulting in a deletion of part of the third transmembrane domain [105].

According to data repositories, at least 15 alternatively spliced transcripts are expressed in humans (Table 2 and Fig. 3). The longest transcripts are ATP13A2-001, ATP13A2-002, and ATP13A2-005. Transcript variants ATP13A2-001 and ATP13A2-005 differ only in a nucleotide segment on exon 5, while transcript variant ATP13A2-002 lacks exons 22 and 28. The ATP13A2 mRNA is highly expressed in the brain, particularly in the *substantia nigra* of patients with classical late-onset PD [91]. However, nothing is known about the splicing expression profiles of this gene in PD and healthy subjects.

The products of these transcripts have been studied at the protein level. The isoform 1 encoded by ATP13A2-001 is a protein of 1180 amino acids with ten transmembrane domains. Isoform 2 encoded by ATP13A2-005 contains a five amino acid deletion near the N-terminus, while isoform 3, encoded by ATP13A2-002, contains two deletions, generating a highly diverged C-terminus [105]. Functional studies have shown that the isoform 1 is located in the lysosome membrane, whereas the isoform 3 protein is retained in the endoplasmic reticulum and rapidly degraded by the proteasome. In addition, both isoforms 1 and 3 are eliminated via the endoplasmic reticulum-associated degradation pathway [105].

##### PLA2G6

Recessive mutations in the *phospholipase A2 group VI* (*PLA2G6*) gene have been initially described as the cause of infantile neuroaxonal dystrophy and neurodegeneration associated with brain iron accumulation. Recently, this gene has also been associated with a particular parkinsonian phenotype, consisting of levodopa-responsive dystonia, pyramidal signs, and cognitive/psychiatric features, with onset in early adulthood [106]. Among *PLA2G6* identified mutations, the c.1077G > A mutation at the last nucleotide of exon 7 (apparently a synonymous mutation) stands out as a cause of abnormal mRNA splicing. This single nucleotide substitution causes the activation of a cryptic splice site producing a 4-bp deleted transcript with altered frame shift in leukocytes [106].

*PLA2G6* gene maps on chromosome 22 (q13.1), covering 70 kb of genomic DNA. Several transcript variants encoding multiple isoforms have been described up to now (Table 2 and Fig. 3). The longest *PLA2G6* mRNA PLA2G6-001 includes 17 exonic regions and encodes the 85/88 kDa calcium-independent phospholipase known as A2 isoform a. The other two long transcripts (PLA2G6-002 and PLA2G6-201) differ in the start point, both lack of exon 9 and encode the same protein, called isoform b. The expression profile of this gene in healthy and disease states remains unknown.

##### FBXO7

Mutations in the *F*-*box only protein 7* (*FBXO7*) gene cause parkinsonian pyramidal disease (PPD- or PARK15-associated parkinsonism), an autosomal recessive neurodegenerative disease with juvenile onset, severe levodopa-response, and additional pyramidal signs. Some pathogenic mutations have been identified (R378G, R498X, and T22M) including a compound heterozygous mutation (IVS7 + 1G/T) that removes the invariable splice donor of intron 7 and may disrupt *FBXO7* messenger RNA splicing [107–109].

The *FBXO7* gene, mapped on chromosome 22q12.3, contains nine exons spanning about 24.1 kb. It encodes a 522 amino acid protein consisting of several domains [108], which target proteins for ubiquitination [108]. Alternatively spliced transcript variants of this gene have been identified (Table 2 and Fig. 3) [107]. FBXO7-001 is the longest and more abundant transcript ubiquitously expressed [110], particularly in skin fibroblasts [111]. FBXO7-002 arises from an inner alternative exon 1, differs in the start codon, and produces a shorter isoform. Both these encoded protein isoforms have been detected in cells [111].

### X-linked parkinsonism

**Fig. 4:**
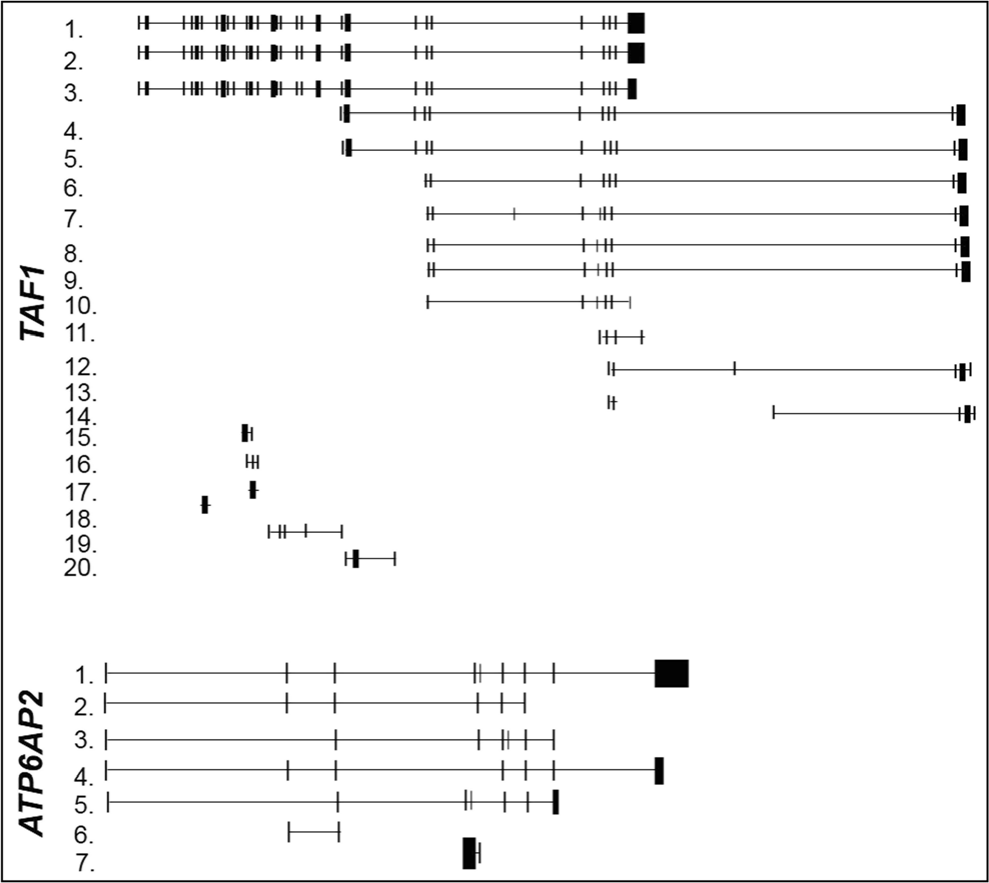
Structures of the alternative splicing variants of human X-linked PD genes. Structures of the described mRNA splicing variants are represented in the figure as reported in Ensembl library (http://www.ensembl.org/index.html). On the *left*, each variant is indicated with a *number* corresponding to that indicated in Table 3. All transcripts are illustrated in 5′-3′ sense

X-linked dystonia parkinsonism (XDP) is an X-linked recessive adult-onset movement disorder characterized by both dystonia and parkinsonism. *TATA*-*box binding protein*-*associated factor 1* (*TAF1*) gene, located in the disease locus Xq13.1, has been reported as the first related XPD gene, harboring disease-specific single-nucleotide changes and a small deletion within the multiple transcript panel [112]. This gene is part of a complex region of DNA (the TAF1/DYT3 multiple transcript systems), which encompasses the exonic regions of TAF1 gene and further additional downstream exons [112, 113]. This system includes multiple different transcription start sites and encodes multiple spliced transcripts and isoforms (Table 3 and Fig. 4) [112].

**Table 3:** Alternative splice variants of human X-linked PD genes

Gene name	Transcript number	Ensembl name	Genbank accession number	Protein length
*TAF1*	1.	TAF1-201	NM_001286074	1895 aa
	2.	TAF1-009	NM_138923	1872 aa
	3.	TAF1-008	NM_004606	1893 aa
	4.	TAF1-014	–	458 aa
	5.	TAF1-010	–	490 aa
	6.	TAF1-021	–	No protein
	7.	TAF1-011	–	No protein
	8.	TAF1-013	–	No protein
	9.	TAF1-012	–	No protein
	10.	TAF1-015	–	No protein
	11.	TAF1-018	–	No protein
	12.	TAF1-016	–	No protein
	13.	TAF1-022	–	No protein
	14.	TAF1-023	–	No protein
	15.	TAF1-005	–	No protein
	16.	TAF1-006	–	No protein
	17.	TAF1-020	–	No protein
	18.	TAF1-019	–	No protein
	19.	TAF1-017	–	279 aa
	20.	TAF1-007	–	150 aa
*ATP6AP2*	1.	ATP6AP2-004	NM_005765	350 aa
	2.	ATP6AP2-007	–	203 aa
	3.	ATP6AP2-005	–	No protein
	4.	ATP6AP2-006	–	243 aa
	5.	ATP6AP2-001	–	259 aa
	6.	ATP6AP2-003	–	No protein
	7.	ATP6AP2-002	–	No protein

Gene name, Ensembl transcript names, GenBank accession numbers, and relative encoded amino acidic protein length of splice variants are reported in the table. The number in the column “Transcript number” identifies the transcript in Fig. 4

Recently, the *ATP6AP2* gene (Table 3 and Fig. 4) has been proposed as a novel gene for X-linked parkinsonism with spasticity (XPDS) by exome sequencing analysis [114]. A silent mutation (p.S115S) in the *ATP6AP2* gene has been identified in one affected individual, resulting in the aberrant splicing of ATP6AP2 mRNA and the overexpression of a minor splice isoform [114]. Noteworthy, the ATP6AP2 is an essential accessory component of the vacuolar ATPase required for lysosomal degradative functions and autophagy, a pathway frequently affected in PD.

### Other PD-related genes

#### SNCAIP

Synphilin-1, encoded by *SNCAIP* gene, is a presynaptic protein containing several protein–protein interaction motifs, including ankyrin-like repeats, a coiled-coil domain, and an ATP/GTP-binding domain [115]. It interacts strongly with alpha-synuclein in neuronal tissue and may play a role in the formation of Lewy bodies during neurodegeneration. It is also implicated in parkinsonism as one of the parkin substrates. In addition, some studies have identified *SNCAIP* sequence variants in PD patients and have suggested it as a candidate PD gene [116, 117].

**Fig. 5:**
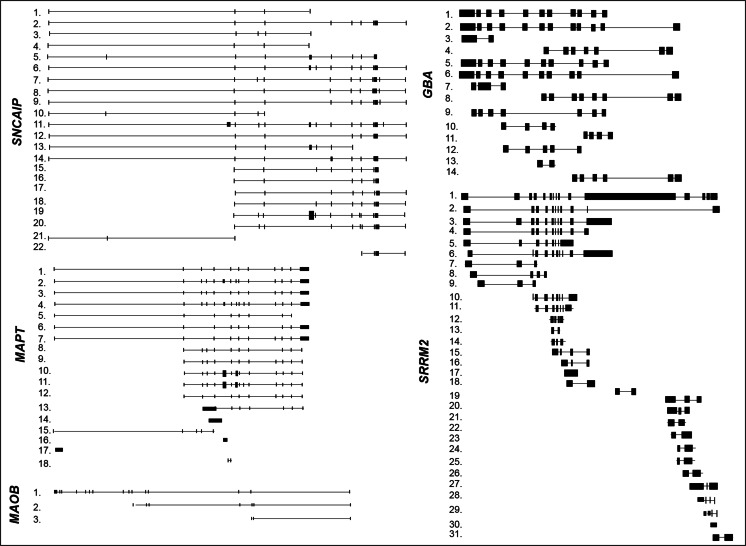
Structures of the alternative splicing variants of the other human PD-related genes. Structures of the described mRNA splicing variants are represented in the figure as reported in Ensembl library (http://www.ensembl.org/index.html). On the *left*, each variant is indicated with a *number* corresponding to that indicated in Table 4. All transcripts are illustrated in 5′-3′ sense, except *MAO*-*B* and *GBA* genes, which are illustrated in antisense corresponding to their 3′-5′ sense transcription

*SNCAIP* gene maps on chromosome 5 (5q23.2) and spans about 152 kb of genomic DNA. Although the database Gene reports *SNCAIP* composed of 11 exons, additional exonic regions emerge by aligning the sequence of the gene with each transcript. To date, at least 22 alternative spliced transcript variants have been identified (Table 4 and Fig. 5), but the most studied are synphilin-1 and 1A. Synphilin-1 (SNCAIP-001) is the full-length transcript, while synphilin-1A variant is a shorter form (SNCAIP-201). The latter lacks exons 4 and 5 and contains an extra exon located between exons 10 and 11. Synphilin-1A isoform is thought to be involved in the pathogenesis of PD and may play an important role in the formation of Lewy bodies [118–120]. Interestingly, synphilin-1A protein shows enhanced aggregation properties, which cause neuronal toxicity [118–120].

**Table 4:** Alternative splice variants of other human PD-related genes

Gene name	Transcript number	Ensembl name	Genbank accession number	Protein length
*SNCAIP*	1.	SNCAIP-019	–	135 aa
	2.	SNCAIP-003	–	66 aa
	3.	SNCAIP-016	–	161 aa
	4.	SNCAIP-017	–	98 aa
	5.	SNCAIP-010	–	858 aa
	6.	SNCAIP-001	NM_005460	919 aa
	7.	SNCAIP-204	–	113 aa
	8.	SNCAIP-201	NM_001242935	603 aa
	9.	SNCAIP-004	–	66 aa
	10.	SNCAIP-018	–	68 aa
	11.	SNCAIP-002	–	1016 aa
	12.	SNCAIP-006	–	62 aa
	13.	SNCAIP-005	–	No protein
	14.	SNCAIP-007	–	66 aa
	15.	SNCAIP-203	–	88 aa
	16.	SNCAIP-202	–	62 aa
	17.	SNCAIP-012	–	66 aa
	18.	SNCAIP-011	–	88 aa
	19.	SNCAIP-009	–	588 aa
	20.	SNCAIP-008	–	113 aa
	21.	SNCAIP-015	–	14 aa
	22.	SNCAIP-013	–	No protein
*MAO*-*B*	1.	MAOB-001	NM_000898	520 aa
	2.	MAOB-002	–	No protein
	3.	MAOB-004	–	No protein
*GBA*	1.	GBA-011	–	No protein
	2.	GBA-001	NM_000157	536 aa
	3.	GBA-002	NM_001005741; NM_001005742	536 aa
	4.	GBA-003	–	No protein
	5.	GBA-009	–	No protein
	6.	GBA-015	NM_001171812	487 aa
	7.	GBA-016	NM_001171811	449 aa
	8.	GBA-005	–	No protein
	9.	GBA-012	–	No protein
	10.	GBA-006	–	No protein
	11.	GBA-010	–	No protein
	12.	GBA-014	–	No protein
	13.	GBA-007	–	No protein
	14.	GBA-013	–	No protein
*MAPT*	1.	MAPT-204	NM_005910	441 aa
	2.	MAPT-202	NM_001123067	412 aa
	3.	MAPT-201	NM_016835	758 aa
	4.	MAPT-205	NM_001203251; NM_001203252	410 aa
	5.	MAPT-203	NM_001123066	776 aa
	6.	MAPT-013	–	No protein
	7.	MAPT-001	NM_016841	352 aa
	8.	MAPT-002	NM_016834	383 aa
	9.	MAPT-006	–	410 aa
	10.	MAPT-007	–	441 aa
	11.	MAPT-008	–	758 aa
	12.	MAPT-004	–	776 aa
	13.	MAPT-003	–	412 aa
	14.	MAPT-009	–	341 aa
	15.	MAPT-014	–	No protein
	16.	MAPT-011	–	59 aa
	17.	MAPT-010	–	No protein
	18.	MAPT-012	–	No protein
*SRRM2*	1.	SRRM2-001	NM_016333	2752 aa
	2.	SRRM2-201	–	311aa
	3.	SRRM2-003	–	1018 aa
	4.	SRRM2-006	–	297 aa
	5.	SRRM2-004	–	No protein
	6.	SRRM2-007	–	895 aa
	7.	SRRM2-011	–	No protein
	8.	SRRM2-028	–	94 aa
	9.	SRRM2-012	–	115 aa
	10.	SRRM2-013	–	251 aa
	11.	SRRM2-029	–	No protein
	12.	SRRM2-014	–	No protein
	13.	SRRM2-030	–	No protein
	14.	SRRM2-015	–	No protein
	15.	SRRM2-016	–	No protein
	16.	SRRM2-017	–	No protein
	17.	SRRM2-009	–	No protein
	18.	SRRM2-018	–	No protein
	19.	SRRM2-019	–	No protein
	20.	SRRM2-022	–	No protein
	21.	SRRM2-020	–	184 aa
	22.	SRRM2-021	–	No protein
	23.	SRRM2-023	–	No protein
	24.	SRRM2-024	–	No protein
	25.	SRRM2-025	–	No protein
	26.	SRRM2-026	–	No protein
	27.	SRRM2-010	–	No protein
	28.	SRRM2-027	–	No protein
	29.	SRRM2-031	–	78 aa
	30	SRRM2-032	–	41 aa
	31.	SRRM2-033	–	No protein

Gene name, Ensembl transcript names, GenBank accession numbers and relative encoded amino acidic protein length of splice variants are reported in the table. Number in the column “Transcript number” identifies the transcript in Fig. 5

The mRNA expression levels of synphilin 1, 1A, and other two additional synphilin variants have been simultaneously investigated in the frontal cortex of PD patients. Their overall overexpression has been demonstrated when compared to healthy controls [24, 65].

#### MAPT

*MAPT* gene encodes the microtubule-associated protein tau, a protein involved in microtubule assembly and stability [121]. It is located on chromosome 17q21 and contains 15 exons. It gives rise to multiple splice transcripts (Table 4 and Fig. 5) which are differentially expressed in human tissues [11]. In the adult human central nervous system, *MAPT* splicing generates six tau isoforms composed of either three or four microtubule-binding repeat motifs in the C-terminal (3R- and 4R-tau).

A number of mutations within and around *MAPT* exon 10 disrupt exonic and intronic splicing elements as well as the formation of an RNA stem-loop structure at the 5′ splice site (which normally functions to restrict spliceosome assembly). This event results in an altered ratio of 3R/4R isoforms [10, 13]. The disruption of the balance between them results in hyperphosphorylation and aggregation of tau proteins into neurofibrillary tangles, causing the frontotemporal dementia with parkinsonism linked to chromosome 17 (FTDP-17) [10, 13]. These data support a direct relationship between aberrant alternative splicing of tau and neuropathology.

#### GBA

Mutations in β-glucocerebrosidase (*GBA*) gene cause Gaucher disease, a lysosomal storage disease characterized by an accumulation of glucocerebrosides. Some studies have identified *GBA* genetic variants as significant risk factors for the development of PD [122, 15].

*GBA* gene is located in a gene-rich region on chromosome 1q21. It spans 10.4 kb and contains 12 exons. Currently, there are four annotated alternative transcripts encoding proteins (GBA-001, GBA-002, GBA-015, and GBA-016; Table 4 and Fig. 5). Two of them originate from an alternative promoter located 2.6 kb upstream of the first ATG [123]. All transcripts share the same start codon, with the exception of GBA-012, whose open-reading frame starts upon exon 4 and produces a shorter protein isoform. Further transcripts are produced, but they do not encode proteins. The *GBA* splicing profile has not been studied still, and it is unknown if its alternative splicing is involved in PD.

#### MAO-B

*MAO*-*B* gene is located on chromosome X and includes 15 exons (Table 4 and Fig. 5). Although it is not a confirmed susceptibility gene [18], increased levels of monoamine oxidase B (MAO) mRNA and enzymatic activity have been reported in platelets from patients with both Parkinson’s and Alzheimer’s diseases [124]. Furthermore, it is well established that MAO-B inhibitors delay progression of both pathologies [125, 126].

Several DNA polymorphisms in the *MAO*-*B* gene have been described in populations with distinct ethnic backgrounds [124]. A SNP common in all ethnic groups and associated with two-fold risk of PD is the G/A dimorphism in intron 13 sequence [127–129]. This SNP does not change the coding sequence and does not affect the consensus acceptor and donor sites. However, it has been demonstrated the G/A dimorphism in intron 13 sequence creates a splicing enhancer that stimulates intron 13 removal and a spliceosomal complex assembly and alters splicing factors’ binding site efficiency [124].

#### SRRM2

Along with *cis*-acting elements, alternative splicing regulation relies on *trans*-splicing factors including the serine/arginine (SR) proteins. One of these proteins, the RNA splicing factor SRRM2 (or serine/arginine repetitive matrix 2), has been identified as the only gene that stood out as differentially expressed in multiple gene expression PD datasets [130].

*SRRM2* gene generates two main alternative splicing transcripts different at their 3′ end (Table 4 and Fig. 5). The full-length isoform SRRM2-001 contains 15 exons, while the shorter isoform SRRM2-003 contains 11 exons and lacks exons 12–15. These two isoforms are differentially expressed in postmortem PD brain regions [130]. The shorter transcript was upregulated in the *substantia nigra* but unchanged in the *amygdala* of PD patients versus healthy controls. On the contrary, the longer transcript was downregulated in both *substantia nigra* and *amygdala* of PDs as compared to controls [130]. Furthermore, in the peripheral blood of patients with PD, SRRM2 short isoform is overexpressed, while the expression of longest isoform is reduced [130].

## Genome-wide RNA expression analysis reveals global alternative splicing changes in PD

Although a “gene-by-gene” approach may simplify splicing analysis, global alternative splicing changes in PD have to be considered. The majority of the whole gene expression array studies in PD brain regions have unfortunately looked at a single transcript per gene, ignoring the multiple transcripts generated by alternative splicing [14]. Nonetheless, mRNA splicing has been identified as a mechanism significantly altered in cortical neurons of PD patients [131].

In order to investigate the splicing expression changes, some studies have used exon arrays. This kind of approach, enabling better monitoring and detection of the alternative splicing events, has allowed to observe significant changes in overall gene splicing in PD blood cells compared to healthy controls [130, 132]. Another exon array study has been conducted in blood of advanced PD patients prior to and following deep brain stimulation neurosurgery, a technique that efficiently improves the motor symptoms of PD [133]. This analysis has showed preliminary results suggesting brain electrical stimulation may correlate with significant profile changes in nonsense-mediated mRNA decay (an mRNA surveillance process that detects and selectively degrades splice transcripts harboring premature termination codons) in blood cell transcripts [133]. Potashkin et al. [134] have also used specific splice variant microarrays in PD patients in order to identify mRNAs splice transcripts as molecular biomarkers for an early PD diagnosis. Through this approach, they identified 13 splice variants with an altered expression in early-stage PD patients versus healthy controls [134, 135].

A recent technology to better study splicing defects is deep sequencing of RNA (RNAseq) [14]. The advantage of RNAseq is that it is theoretically feasible to measure both RNA expression levels and modifications such as splicing. In addition, RNAseq gives the possibility of discovering novel transcripts. Whole transcriptome RNAseq data have been obtained from blood leukocytes of PD patients’ predeep and postdeep brain stimulation treatment [136]. This approach has enabled to discover novel human exons and junctions in protein-coding RNA molecules, as well as a large range of differential splicing events pretreatment and posttreatment compared to healthy controls [136]. Although this is the first study using in-depth PD transcriptome sequencing, RNAseq represents a promising technique to better study PD alternative splicing.

## The role of miRNA and lncRNA in PD alternative splicing modulation

A large number of alternative exon regions have been predicted as binding sites of microRNAs (miRNAs). The latter is a class of small noncoding RNA molecules, which mainly act as posttranscriptional modulators of multiple target genes by partial sequence complementarity. Through this mechanism, they may also influence splicing process.

The interplay between miRNA differential expression and alternative splicing modification in PD has been recently investigated [137]. Parallel changes in miRNA profiles and their spliced targets have been observed in PD leukocytes and PD-relevant brain regions (including the *substantia nigra* as well as the *frontal lobe*). This study was conducted through coupled analysis of small RNA sequencing data, splice junction arrays, and exon arrays [137].

Another novel fascinating class of RNAs with unknown functions is long noncoding RNAs (lncRNAs), defined as transcripts of over 200 nucleotides. The GENCODE noncoding RNA set collects all lncRNAs known so far, including several spliced transcript shorter than 200 bp. LncRNA profiling has been recently assessed in PD leukocytes predeep and postdeep brain stimulation via RNAseq [136]. This survey allowed to identify some lncRNAs overexpressed in PD and inversely decreased following deep brain stimulation [136]. Differentially expressed lncRNA includes the spliceosome component U1, supporting the hypothesis of disease-involved splicing modulations [136].

The identification of existing networks between noncoding mRNAs and alternative splicing modifications represents an important step forward the road to understanding the molecular basis of PD.

## Conclusions

Alternative splicing is a highly harmonized process, based on a combination of DNA sequence motifs, intronic and exonic elements, regulatory factors, and temporal and spatial signaling pathways. Mutations that disrupt any of these critical features may alter the finely tuned splicing processes, upsetting the production or functions of the encoded proteins, and finally causing human diseases. Assessing the alternative splicing modulation of PD-related genes represents an important point to understand PD molecular etiology. Future studies, both with the standard or the new currently available large-scale techniques, will offer a complete data pool of the alternative splicing events in PD and will provide new possible insights in order to develop strategies for PD therapy and diagnosis.
